# Prevention and Treatment of Cardiometabolic Diseases in Children with Overweight and Obesity: The Future of Healthcare

**DOI:** 10.3390/children9020176

**Published:** 2022-02-01

**Authors:** Valeria Calcaterra, Gianvincenzo Zuccotti

**Affiliations:** 1 Department of Pediatrics, “Vittore Buzzi” Children’s Hospital, 20157 Milano, Italy; gianvincenzo.zuccotti@unimi.it; 2 Pediatric and Adolescent Unit, Department of Internal Medicine, University of Pavia, 27100 Pavia, Italy; 3 Department of Biomedical and Clinical Science “L. Sacco”, University of Milano, 20157 Milano, Italy

**Keywords:** complications, cardiovascular, metabolic, obesity, children, adolescents, weight-related diseases

## Abstract

In this Special Issue we will consider the impact of obesity on health in order to review the latest findings on the risk factors associated with cardiometabolic diseases in children with overweight and obesity as well as to explore the pathogenic mechanisms and potential therapeutic targets. The role of weight-management strategies, including exercise, dietary changes and nutritional education, in preventing obesity-related complications will be considered. The improvement of many obesity-associated complications following bariatric surgery will also be reported. The timely implementation of preventive strategies in pediatric patients with overweight and obesity may ameliorate the future burden of weight-related diseases and the future of healthcare.

Pediatric obesity represents a significant issue of public health [1,2,3]. According to the World Health Organization (WHO), 40 million children aged < 5 years in addition to more than 330 million subjects aged 5–19 years were overweight or obese [2], with severe adverse consequences for the wellbeing of individuals and associated costs for society [3,4].

Childhood obesity is a result of genetics, environmental factors and developmental influences in addition to the complex interactions between them [1]. Pediatric obesity leads to complications and comorbidities during childhood and adolescence that persist into adulthood, affecting almost every system in the body [4,5] and predicting a shorter life expectancy [4,5]. In the short term, children with obesity suffer from cardiometabolic risk factors [6], such as high blood pressure, dyslipidemia, type two diabetes, abnormalities of the cardiovascular system, respiratory disease, low-grade systemic inflammation, liver disorders and musculoskeletal problems. Obese children also show psychological problems, including depression, anxiety, low self-esteem and behavioral disorders. In the long term, obesity during childhood increases the risk of developing cardiovascular diseases, diabetes and some cancers in adulthood that lead to premature death [1,4,7].

The spectrum of cardiometabolic disorders begins with relative insulin resistance (IR). IR is expressed early in life, progressing from prediabetes and metabolic syndrome to T2DM or CVD [1,2,6,7]. Starting in pediatric ages, the chronic inflammatory status associated with obesity plays a relevant role in the development of complications [8]. Obesity leads to the increased secretion of proinflammatory cytokines and inflammatory markers, the dysregulated secretion of adipocytokines in addition to the infiltration and dysfunction of immune cells. This process may facilitate a state of low-grade inflammation, which may be a pivotal mechanism linking obesity to its metabolic and cardiovascular complications [8].

The initiating events, including metabolic and cardiovascular derangement, in obesity-induced inflammation start early in childhood, and the adverse effects of obesity on cardiometabolic health in adult ages are independent from adult weight, suggesting the fundamentality of prevention [1,2].

The promotion of an active lifestyle alongside the modification of behavioral movements [9], dietary changes and nutritional education are the best non-pharmacological approaches to reduce the cardiovascular risk. However, in selected cases with severe obesity, a medical treatment or surgery might be useful to prevent cardiometabolic disorders. 

Considering that the acquisition of a healthy lifestyle could track to later ages, a proposal of a preventive approach starting in pediatric ages could preserve children’s health [10]. The early acquisition of an unhealthy lifestyle could act on adult health through different pathways; a “metabolic programming pathway” could cause a synergistic deleterious effect on cardiometabolic health later on [10].

Early childhood education is one of the best investments a country can make to prepare children for learning and give them a chance to thrive later in life. The timely implementation of preventive strategies targeted at reducing the prevalence of obesity during the early years of life can also address health and economic burdens in adult ages [11].

In this Special Issue we will consider the impact of obesity on health in order to review the latest findings on the risk factors associated with cardiometabolic diseases in pediatric subjects with overweight and obesity in addition to explore the pathogenic mechanisms and potential preventive as well as therapeutic targets. The relevant role of weight-management strategies, including behavioral movements, dietary changes and nutritional education, in preventing obesity-related complications will be considered. The improvement of many obesity-associated complications following bariatric surgery will also be reported. Preventive interventions in pediatrics must be proposed in order to ameliorate the future burden of weight-related diseases and the future of healthcare.

## Data Availability

Not applicable.
